# Changes in Motor, Functional Independence, and Gait Recovery After Incomplete Spinal Cord Injury with Transcutaneous Spinal Cord Stimulation: A Randomized Controlled Trial with a Partial Crossover Design

**DOI:** 10.3390/biomedicines14061214

**Published:** 2026-05-27

**Authors:** Hatice Kumru, Aina Ros-Alsina, Agustín Hernandez-Navarro, Eloy Opisso, Margarita Vallès, Jesus Benito-Penalva, Joan Vidal, Miquel Sarrio, Loreto García-Alén

**Affiliations:** 1 Guttmann Hospital de Neurorehabilitació, Institut Universitari Adscrit a la Universitat Autònoma de Barcelona, 08916 Badalona, Spain; ainarosalsina@gmail.com (A.R.-A.); ahernandez@guttmann.com (A.H.-N.); eopisso@guttmann.com (E.O.); mvalles@guttmann.com (M.V.); jbenito@guttmann.com (J.B.-P.); jvidal@guttmann.com (J.V.); msarrio@guttmann.com (M.S.); loretogarcia@guttmann.com (L.G.-A.); 2 Institut de Recerca Germans Trias i Pujol, 08916 Badalona, Spain; 3 Department of Automatic Control, Escola Tècnica Superior d’Enginyeria Industrial de Barcelona (ETSEIB), Universitat Politècnica de Catalunya—BarcelonaTech (UPC), Diagonal 647, 08028 Barcelona, Spain

**Keywords:** multisegmental transcutaneous spinal cord stimulation, repeated sessions, spinal cord injury, muscle strength, TUG, SCIM-III, gait

## Abstract

**Background:** Transcutaneous spinal cord stimulation (tSCS) is a promising approach to enhance functional recovery after spinal cord injury (SCI). However, evidence on repeated sessions and their effects on gait and lower-limb strength remains limited. This study evaluated the effects of multisegmental tSCS on walking ability, muscle strength, and functional independence in individuals with SCI. **Methods:** In this randomized controlled trial with a partial crossover design, twelve individuals received tSCS combined with gait rehabilitation, while ten underwent gait rehabilitation alone, for three weeks. Four participants crossed over to the tSCS group after a minimum one-week washout period following the control intervention. We assessed the American Spinal Injury Association Impairment Scale (AIS), Total Motor Score (TMS), Lower Extremity Motor Score (LEMS), Walking Index for Spinal Cord Injury II (WISCI-II), 10- and 6-Meter Walking Tests (10MWT, 6meterWT), Timed Up and Go (TUG) test, maximal voluntary contraction (MVC) of the quadriceps (QM) and tibialis anterior (TA), and the Spinal Cord Independence Measure (SCIM-III); tSCS was applied at three spinal segments during gait rehabilitation over 15 sessions. **Results:** tSCS significantly improved MVC in both muscles, as well as SCIM-III and TUG, and these improvements were maintained at follow-up, with no significant adverse events reported. Other clinical assessments also showed significant improvement in both groups. **Conclusions:** tSCS was well tolerated and conferred additional benefits in lower-limb muscle strength, walking ability (as assessed by TUG), and functional independence, supporting its potential as a valuable adjunct to rehabilitation.

## 1. Introduction

Spinal cord injuries (SCIs) are complex medical conditions that may result from traumatic or non-traumatic causes and often lead to severe morbidity and permanent disability. The degree of gait impairment depends on both the severity and location of the injury [1,2,3,4,5]. The loss of independent walking markedly reduces quality of life by limiting mobility, autonomy, and social participation [2,3,4,5]. In incomplete SCI, however, residual neural connectivity across the lesion provides an opportunity for functional recovery. Beyond mobility limitations, impaired ambulation contributes to secondary complications such as muscle atrophy, joint contractures, bone demineralization, cardiovascular deconditioning, and metabolic dysfunction [2,3,4,5]. Thus, restoring gait is not only a functional goal but also a crucial strategy to prevent secondary health issues in the long-term management of SCI.

Neuroplasticity, the ability of the nervous system to reorganize and form new connections, plays a central role in recovery after SCI. Non-invasive neuromodulation techniques, such as spinal cord stimulation (SCS), and particularly transcutaneous spinal cord stimulation (tSCS), have gained increasing attention for their potential to improve motor outcomes [6,7,8,9,10,11,12,13,14,15,16,17]. The central hypothesis of tSCS is that it modulates spinal sensorimotor networks above, within, and below the lesion, thereby facilitating voluntary motor control by elevating these networks to a more excitable functional state [11,12]. Repeated tSCS sessions may induce activity-dependent plasticity, leading to long-term reorganization of spinal circuits. Such plasticity can reinforce learned motor functions, which may persist even after stimulation ends [7,15,16,17,18,19,20,21,22,23,24,25,26].

Several studies have evaluated the effects of single or repeated sessions of tSCS on gait function following SCI. Overall, studies examining the effects of single or repeated tSCS sessions on gait function after SCI have predominantly used single-site stimulation at the T11 level in individuals with incomplete injuries, in combination with different gait rehabilitation approaches, including treadmill stepping [6,24], robot-assisted gait training [7,13,23], exoskeleton-assisted walking [22], mobilization [12], and conventional gait training [25].

Evidence suggests that multisegmental stimulation may more effectively engage locomotor networks. Studies have demonstrated that multisite tSCS can induce coordinated stepping movements in healthy individuals and rhythmic leg movements in people with SCI [11,12]. In addition, combined cervical transcutaneous and lumbosacral epidural stimulation has been shown to enhance voluntary control of stepping in individuals with chronic motor complete paralysis [27]. A synergistic effect has also been reported when tSCS at Coc1 and/or T11 was paired with exoskeleton-assisted therapy in an individual with SCI [15].

The rationale for multisegmental stimulation is supported by the intrinsic rhythmogenic properties of the cervico-lumbosacral spinal cord. Cervical tSCS may facilitate remote neuromodulation, increasing cortical excitability and modulating cortical responsiveness [21,22,28], as well as influencing lower-limb spinal reflex circuits in healthy individuals [29]. The lumbar cord is particularly well suited for generating bursting activity and locomotor patterns, whereas the sacral cord contributes to rhythm generation via the activation of afferent inputs, motor axons, and excitatory ascending propriospinal pathways projecting to lumbar locomotor networks [30,31,32].

To date, only three studies have examined the effects of repeated tSCS sessions on gait recovery. Two studies involved individuals with subacute incomplete SCI (<6 months post-injury) and included sham or control conditions [7,23], whereas one study in individuals with chronic SCI did not include a control group [25]. All three studies delivered stimulation at the T11 spinal segment. More recently, multisegmental tSCS (cervical, lumbar, and coccygeal spinal segments) applied during gait training was shown to produce greater improvements in walking speed and lower-limb muscle strength than stimulation delivered to one or two spinal segments alone [9].

Here, we hypothesized that repeated sessions of multisegmental tSCS combined with gait training would enhance the efficacy of the intervention by promoting re-engagement of spinal networks, thereby improving functional independence, lower-limb muscle strength, and walking function in individuals with incomplete SCI, with benefits that translate into sustained functional gains.

## 2. Materials and Methods

The inclusion criteria were as follows: (1) male or female individuals aged 18 years or older; (2) individuals with a stable traumatic or non-traumatic incomplete motor cervical or thoracic spinal cord injury (SCI); (3) time since SCI of 3 months or longer; (4) an American Spinal Injury Association Impairment Scale (AIS) score of C or D [33]; (5) candidates for gait rehabilitation, either in using the Lokomat (Hocoma, Volketswil, Switzerland) or through overground gait therapy; (6) capacity to provide informed consent.

The exclusion criteria were as follows: (1) unstable medical conditions (cancer, acute infections, etc.); (2) severe spasticity (≥3 on the Modified Ashworth Scale (MAS)); (3) peripheral nerve affectation; (4) ulcers at the electrode application site; (5) intolerance to tSCS.

The protocol was approved by the Ethics Committee of the Unió Catalana d’Hospitals’ under the code number “CEI 23/16” and was carried out in accordance with the standards of the Declaration of Helsinki. Informed consent was obtained from all individuals involved in the study. This study was registered at ClinicalTrials.gov (Identifier: NCT07289191).

### 2.1. Outcome Measures

Clinical assessments included neurological and gait assessments. The American Spinal Injury Association (ASIA) Impairment Scale (AIS) was used to evaluate motor and sensory deficits [33]. The AIS classifications were as follows: A—complete sensory and motor SCI; B—incomplete sensory and complete motor SCI; C and D—incomplete sensory and motor SCI. The Total Motor Score (TMS) and Lower Extremity Motor Score (LEMS) were calculated for each subject [33], along with the Spinal Cord Independence Measure (SCIM-III).

**Figure 1:**
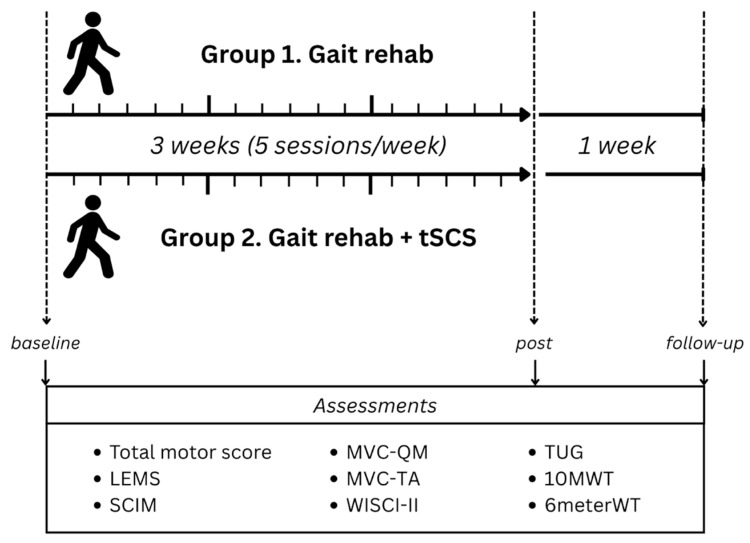
Study conditions and clinical outcome assessments.

Gait function was assessed using the following measures: (i) Walking Index for Spinal Cord Injury II (WISCI-II); (ii) Timed Up and Go (TUG) test; (iii) 10-Meter Walking Test (10MWT); (iv) 6-Meter Walking Test (6meterWT) (Figure 1). The 6meterWT was included to complement the Timed Up and Go (TUG) and 10-Meter Walking Test (10MWT), considering that some individuals with SCI may be unable to complete the 10MWT (Figure 1).

Maximum voluntary contraction (MVC) was assessed in the quadriceps muscle (MVC-QM) and tibialis anterior muscle (MVC-TA).

**Figure 2:**
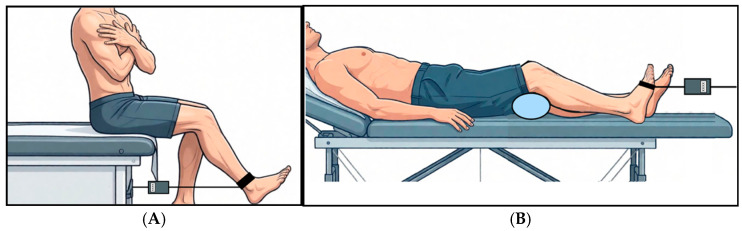
The individual’s posture during the assessment of maximal voluntary contraction (MVC) of (**A**) the quadriceps muscle (MVC-QM) and (**B**) the tibialis anterior muscle (MVC-TA).

For MVC-QM, individuals were seated with a dynamometer positioned parallel to the ground and secured to the ankle of the most affected leg. If both legs were equally affected, the right ankle was selected (Figure 2A).

For MVC-TA, individuals were positioned semi-supine on a bed, with their back reclined at 45 degrees and the knee flexed at 30 degrees, supported by a pillow. A dynamometer was secured at the level of the metatarsophalangeal joint, aligned with the leg and positioned parallel to the ground (Figure 2B).

Individuals initiated a maximal sustained muscle contraction in the quadriceps (QM) and then the tibialis anterior (TA) muscle upon receiving an imperative signal, while electrical stimulation was applied to the wrist at the perception threshold, as determined by the EMG system (Medelec Synergy, Cardinal Health, Surrey, UK). In both the MVC-QM and MVC-TA tests, the highest recorded force sustained for four seconds was considered for analysis, with each test repeated twice.

All clinical assessments were conducted at three time points: baseline (pre), after the final session (post), and one week after the last session (follow-up) (Figure 1).

### 2.2. Study Conditions

This study included two groups: (i) a control group that underwent gait training alone, and (ii) a tSCS group that received tSCS during gait training (Figure 1).

The study was initially designed as a randomized controlled trial, with individuals assigned to either the control or the tSCS group using a computer-generated randomization list. However, participants in the control group were allowed to cross over to the tSCS group upon completion of their follow-up period, upon request. A minimum one-week washout period was required before initiating tSCS in these crossover individuals.

Gait Training: All participants underwent gait training tailored to their individual functional capacity, either on a treadmill or with the assistance of the Lokomat robotic gait orthosis, under the continuous supervision of an experienced physiotherapist to ensure safety and optimal performance. Sessions lasted 30 min and were conducted five days per week for three weeks (15 sessions in total). Participants allocated to the tSCS group received spinal cord stimulation concurrently with gait training, allowing stimulation to be delivered in real time during locomotor practice.

**Figure 3:**
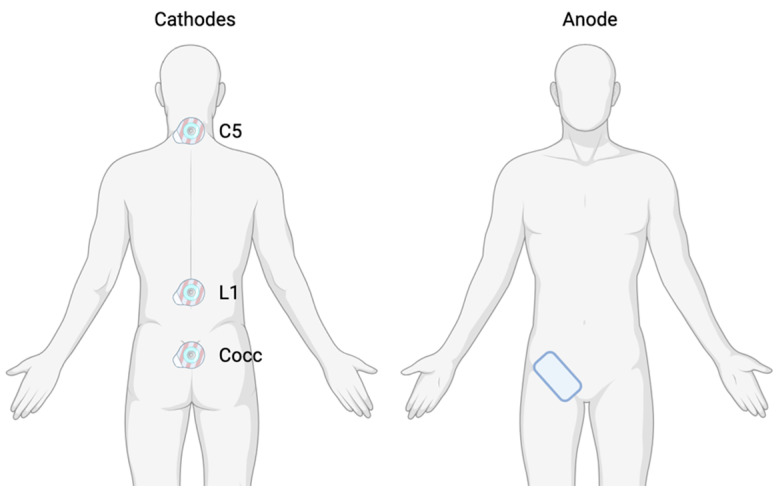
The tSCS electrode placements: C—cervical; L—lumbar; Cocc—coccyx.

tSCS: Stimulation was delivered using the NeoStim-5 transcutaneous electrical stimulator (Co-syma Inc., Moscow, Russia), used solely for research in a clinical setting to evaluate the proof of concept of multisegmental tSCS, rather than to validate a commercial device. The stimulator (anodic-first) was delivered as a 10 kHz carrier with a 1 ms burst width at 30 Hz. Circular hydrogel adhesive electrodes (2 cm diameter; axion GmbH, Hamburg, Germany) served as cathodes, while one rectangular electrode (5 × 12 cm^2^) placed over the iliac crests served as the anode (Figure 3).

Each stimulation channel was configured independently. For each individual, the stimulation intensity was determined as the highest tolerated level at each stimulation site (Table 1, Figure 3). This intensity was identified one day before the intervention and then applied consistently across three weeks of stimulation (15 sessions) during gait training.

tSCS was administered concurrently with gait therapy for a total session duration of 30 min. Before each session, the stimulation intensity was gradually increased over several minutes to allow the individuals to adapt. In addition to gait training, the rehabilitation program included 4–5 h of daily therapy tailored to each individual’s needs. This comprehensive regimen comprised occupational therapy, bipedal standing, hydrotherapy, trunk and core stabilization, upper- and lower-limb strengthening, stretching, and balance and coordination training. All sessions were supervised by a multidisciplinary team to optimize functional recovery and support overall rehabilitation goals.

### 2.3. Data Analysis and Statistics

The TUG and 10MWT were performed once per individual due to fatigue in individuals with SCI, whereas the 6meterWT, MVC-QM, and MVC-TA were conducted twice, and the mean of the two trials was used for statistical analysis.

Measurements were obtained at three time points—baseline (pre), immediately after the final session (post), and one week after the final session (follow-up)—for both the tSCS and control groups (Figure 1).

Data distribution was assessed using the Kolmogorov–Smirnov test. As most variables were not normally distributed, the results are reported as medians and interquartile ranges (IQRs). The Friedman test was used to assess repeated measures across time points, with post hoc Wilcoxon signed-rank tests applied when significant.

Effect sizes were calculated for both neurological and clinical assessments and interpreted as follows: 0.2 = small, 0.5 = medium, and 0.8 = large.

For between-group comparisons, score changes were calculated from baseline (pre) to post-intervention and follow-up. Differences in these changes between groups were analyzed using the Mann–Whitney U test.

To determine whether the effects of tSCS on neurological, functional, and gait outcomes were influenced by baseline characteristics, Spearman’s correlation analyses were conducted. Associations were examined between demographic and baseline variables—including age, time since SCI, baseline TMS and LEMS scores, and stimulation intensity—and changes in clinical outcomes, including LEMS, TMS, SCIM-III, maximal voluntary contraction, and gait performance measures.

An alpha level of 0.05 was set for all comparisons to determine statistical significance.

## 3. Results

**Figure 4:**
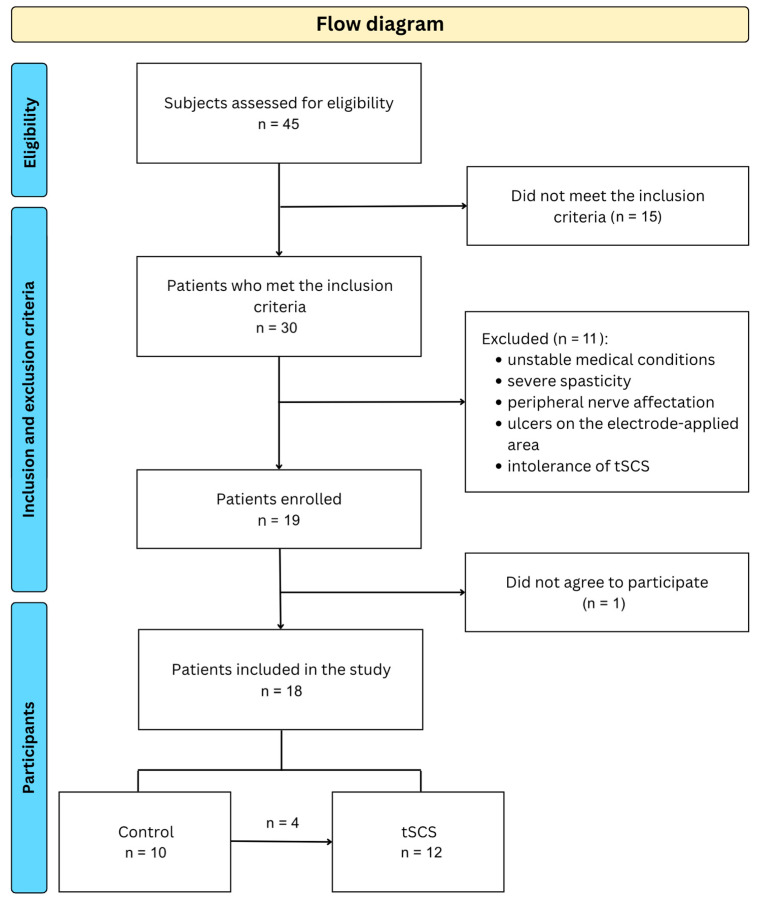
Flow diagram.

This study was a randomized controlled trial with a crossover component, characterized by a partial crossover rate from control to intervention (Figure 4). After providing written informed consent, ten individuals with SCI were assigned to the control group, and eight to the tSCS group. Four SCI individuals participated first in the control group and then in the tSCS group. All individuals had cervical SCIs, except for one with a high thoracic SCI. All individuals completed the study without complications. All individuals were able to perform overground gait rehabilitation, except for one who required robot-assisted training using the Lokomat (Hocoma, Volketswil, Switzerland).

The clinical and demographic characteristics of individuals with SCI are presented in Table 1. The mean age and time since injury were comparable between the two groups (*p* > 0.05). The mean age was 46.7 ± 11.4 years in the control group and 41.8 ± 15.1 years in the tSCS group (*p* = 0.41; Table 1). The time since SCI was 7.5 ± 6.4 months in the control group and 11.2 ± 19.3 months in the tSCS group (*p* = 0.54; Table 1).

The mean tolerated intensity of tSCS at C5 was 41.8 mA (SD 11.2), at L1 was 53.6 mA (SD 7.9), and at Coc1 was 55.8 mA (SD 7.0). The stimulation intensities for each individual and each site are presented in Table 1.

### 3.1. Clinical Assessments

Descriptive statistics (median and IQR) for the Total Motor Score (TMS), Lower Extremity Motor Score (LEMS), and Spinal Cord Independence Measure III (SCIM-III) across all time points (pre, post, and follow-up) and in both groups (control and tSCS) are presented in Table 2 (the individual values can be found in Supplementary Materials, Table S1).

The TMS improved in both the tSCS and control groups (Friedman test: *p* < 0.001 for both groups). Post hoc analysis showed significant improvements after the last session in both the tSCS and control groups (Wilcoxon test: *p* < 0.05 for all comparisons) compared to baseline, which were maintained at follow-up in both groups (Wilcoxon test: *p* < 0.05 for all comparisons) (Table 3).

The LEMS also improved in both the tSCS and control groups (Friedman test: *p* < 0.001 for both groups), with significant post-intervention increases relative to baseline that were maintained at follow-up (Wilcoxon test: *p* < 0.05 for all comparisons in both groups) (Table 3).

Both groups demonstrated moderate effect sizes for TMS and LEMS at both post-intervention and follow-up, indicating meaningful improvements in motor recovery (effect size > 0.5) (Table 3).

SCIM-III showed a significant improvement in the tSCS group (Friedman test: *p* < 0.001). Post hoc analysis revealed a significant improvement after the final session (Wilcoxon test: *p* = 0.018), which was maintained during the follow-up period (Wilcoxon test: *p* = 0.012). In contrast, the control group’s SCIM-III did not change significantly (Friedman test: *p* = 0.210) (Table 3).

In SCIM-III, the tSCS group demonstrated consistently moderate effect sizes at both post-intervention and follow-up, indicating substantial and sustained functional gains. In contrast, the control group showed only small effect sizes, reflecting no improvement (Table 3).

### 3.2. Maximal Voluntary Contraction of Quadriceps (QM) and Tibialis Anterior (TA) Muscles

Table 4 (Table S2 in Supplementary Materials for individual values) present descriptive statistics (median and IQR) for MVC-QM and MVC-TA across all time points (pre, post, and follow-up) and in both groups (control and tSCS).

In the tSCS group, significant improvements were observed in quadriceps and tibialis anterior muscle strength (Friedman test: *p* = 0.001 for QM; *p* < 0.001 for TA). Post hoc comparisons showed significant differences between pre- and post-intervention, as well as between pre-intervention and follow-up, for both muscles (Wilcoxon test: QM, *p* = 0.005 post-intervention and follow-up; TA, *p* = 0.004 after the last session and *p* = 0.002 at follow-up) (Table 3). In contrast, the control group did not show significant changes in either muscle (Friedman test: *p* = 0.14 for QM and *p* = 0.139 for TA) (Table 3).

Overall, the effect sizes were moderate in the tSCS group (effect size > 0.5) for MVC-QM and MVC-TA at post-intervention and follow-up, while the control group showed only small effects (effect size = 0.2 for both muscles) (Table 3).

### 3.3. Gait Assessments

For the Walking Index for SCI II (WISCI-II), Timed Up and Go (TUG), 10-Meter Walking Test (10MWT), and 6-Meter Walking Test (6meterWT), individual values and descriptive statistics (median and IQR) across all time points (pre, post, and follow-up) and in both groups (control and tSCS) are shown in Table 5 (the individual values can be found in Supplementary Materials, Table S3).

**Figure 5:**
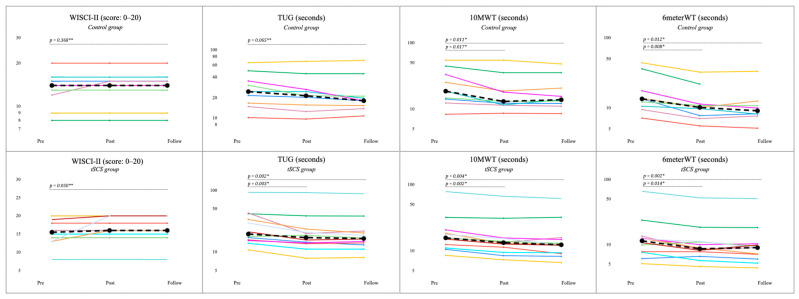
Gait assessments measured using the Walking Index for SCI (WISCI-II), Timed Up and Go (TUG), 10-Meter Walking Test (10MWT), and 6-Meter Walking Test (6meterWT). Each line represents an individual with SCI, and the black dashed line with a circular marker indicates the group median; * *p*-values according to the Wilcoxon signed-rank test for comparisons between pre- and post-intervention, and between pre- and follow-up conditions, in both the control and tSCS groups; ** *p*-values from the Friedman test for non-significant results in the WISCI-II for both groups, and in TUG for the control group.

In the tSCS group, gait function measured by the WISCI-II showed a borderline significant improvement (Friedman test: *p* = 0.05); however, post hoc analysis revealed no significant differences at post-intervention (Wilcoxon test: *p* = 0.109) or at follow-up (*p* = 0.109) (Figure 5). The effect size was small post-intervention and during follow-up (Table 5). Gait function in the tSCS group, assessed using the TUG, 10MWT, and 6meterWT, demonstrated significant improvements (Friedman test: *p* < 0.001 for all assessments). Post hoc analysis revealed significant improvements in the TUG (Wilcoxon test: *p* = 0.003 post-intervention and *p* = 0.002 at follow-up), 10MWT (*p* = 0.002 post-intervention and *p* = 0.004 at follow-up), and 6meterWT (*p* = 0.014 post-intervention and *p* = 0.002 at follow-up) (Table 3, Figure 5). The effect size (effect size > 0.5) was moderate for all gait assessments made post-intervention and during follow-up (Table 3).

In the control group, no significant changes in gait function were observed as assessed by the WISCI-II (Friedman test: *p* = 0.368) or TUG (*p* = 0.065). The effect size was small for the WISCI-II and TUG (effect size < 0.5) (Table 3, Figure 5). Significant improvements were observed only in the 10MWT and 6meterWT (Friedman test: *p* = 0.02 and *p* = 0.002, respectively). Post hoc analysis revealed significant improvements in the 10MWT post-intervention (*p* = 0.017) and maintained at follow-up (*p* = 0.011), and in the 6meterWT (*p* = 0.008 post-intervention and *p* = 0.012 at follow-up) (Table 3). The effect sizes were moderate (effect size > 0.5) for both outcomes at post-intervention and follow-up (Table 3).

### 3.4. Differences Between Groups: Score Changes

Between-group comparisons showed greater improvements in maximal TA muscle strength in the tSCS group at post-intervention and in SCIM-III at follow-up; however, these differences did not reach statistical significance (*p* = 0.06).

At follow-up, score changes in maximal muscle strength were significantly greater in the tSCS group compared to the control group for both MVC-QM (Mann–Whitney U test, *p* = 0.041) and MVC-TA muscle strength (*p* = 0.023) (Table 6).

No significant between-group differences were identified for the TMS, LEMS, WISCI-II, TUG, 10MWT, or 6-meterWT, either immediately post-intervention or at follow-up (Mann–Whitney U test, *p* > 0.05 for all comparisons) (Table 6).

### 3.5. Correlation Analysis in the tSCS Group

Lower baseline LEMS scores were significantly associated with greater improvements in LEMS both immediately post-intervention (ρ = −0.913, *p* < 0.001) and at follow-up (ρ = −0.940, *p* < 0.001), as well as with greater improvements in TMS post-intervention (ρ = −0.664, *p* = 0.018). Interestingly, lower baseline LEMS scores were also strongly associated with faster performance on the 6-Meter Walking Test immediately after the final tSCS session and at follow-up (ρ = 0.586, *p* = 0.045; ρ = 0.687, *p* = 0.014, respectively).

No significant relationships were observed between other baseline characteristics (age, time since SCI, TMS), stimulation intensities, and other clinical outcomes in the tSCS group.

### 3.6. Adverse Effects

All participants included in the study completed the intervention protocol without severe adverse events and demonstrated good tolerance throughout the sessions. Furthermore, no screened participants were excluded due to intolerance to tSCS prior to study inclusion.

Mild undesired effects were reported in participants receiving tSCS (100%; n = 12), but these did not require discontinuation of treatment. Of those, the most reported symptoms were discomfort/mild pain (burning, needles, or pricking sensation) under the electrodes during tSCS at the cervical segment (100%; n = 12) and at the coccyx segment (92%; n = 11), and less frequently at the lumbar segment (58%; n = 7), as well as transitory skin redness after tSCS (92%; n = 11) at any level.

## 4. Discussion

The present randomized controlled trial with a partial crossover component investigated repeated multisegmental tSCS combined with rehabilitation in individuals with incomplete SCI. Both groups improved in general motor outcomes; however, tSCS provided additional benefits in muscle strength, functional independence, WISCI-II, and TUG. tSCS was well tolerated, with only mild, transient adverse effects, supporting its safety and feasibility.

Both groups showed significant improvements in TMS and LEMS with moderate effect sizes at follow-up, indicating overall motor recovery and the contribution of conventional rehabilitation in subacute SCI. The absence of between-group differences may be due to the limited sensitivity of clinical motor scales to detect subtle or task-specific changes.

In contrast, SCIM-III improved significantly only in the tSCS group, with sustained moderate effect sizes, suggesting greater functional recovery than rehabilitation alone. Similarly, maximal voluntary contraction of lower-limb muscles improved only in the tSCS group, with greater between-group gains at follow-up. These findings support the role of tSCS in enhancing voluntary muscle activation via increased spinal excitability and recruitment of residual pathways.

Gait outcomes showed a mixed pattern. The tSCS group improved across all gait measures (WISCI-II, TUG, 10MWT, 6meterWT), whereas the control group improved only in the 10MWT and 6meterWT. These results suggest that tSCS may benefit more complex mobility tasks such as the TUG, which require coordination and balance [34].

Between-group differences in this study were limited, with significance observed only in muscle strength at follow-up. This may be due to the small sample size, heterogeneity, and/or the short duration of the intervention.

Few studies have investigated repeated tSCS combined with gait training. One study included a control group [7], another included a sham condition [23], and one study had no control group [25]. One report also described a single case with pharmacological co-intervention [15]. Repeated tSCS using single-site T11 stimulation has been shown to improve motor strength and gait function in subacute SCI (<6 months). These effects were observed after 8 days of gait training [7] and after 20 days of Lokomat-assisted training [23]. Long-term effects appear to be dependent on cumulative stimulation dose and integration with rehabilitation. In contrast, McHugh et al. [25] reported gait improvements after 23 sessions in chronic incomplete SCI, albeit without a control group.

Most studies used single-site tSCS at T11, combined with treadmill training [6,24], robotic gait training [7,13,23], mobilization [12], or exoskeleton use [35]. Gad et al. [15] used stimulation at T11 and/or Coc1 during exoskeleton therapy. Some studies were limited to single sessions or case reports [13,24]. Stimulation intensity was usually set at sensory threshold or maximum tolerable levels [7,23,24,25] or adjusted individually [15].

tSCS likely acts by modulating spinal excitability via posterior root afferents [6,8,10,11,12,13]. Repeated stimulation combined with training promotes activity-dependent neuroplasticity [7,10,16,17]. The multisegmental approach (cervical, lumbar, sacral) may enhance network engagement for posture and locomotion [9]. Mechanistically, tSCS increases spinal network excitability without directly eliciting action potentials, modulating interneuronal circuits and possibly engaging cutaneous afferents [6,8,10,11,12,13,14]. Multilevel stimulation can induce coordinated stepping in healthy individuals [11] and rhythmic leg activity in individuals with SCI [12]. These effects may contribute to improvements in walking performance and muscle force [9]. Combined cervical, lumbar, and coccygeal stimulation may enhance strength and gait recovery, while cervical stimulation can increase cortical excitability and modulate reflexes [9,21,22,28,29]. Synergistic effects have been reported with Coc1/T11 stimulation and exoskeleton therapy [15]. The lumbosacral cord exhibits strong rhythmogenic properties [30,31], with sacral circuits contributing via afferent activation and propriospinal pathways [32].

Another finding of this study is that lower baseline LEMS scores were associated with greater improvements in motor strength and gait outcomes measured by the 6meterWT following tSCS. This suggests that individuals with more severe impairments may have greater potential for recovery following tSCS, possibly due to reduced ceiling effects.

Several limitations should be acknowledged. The partial crossover design may have affected group allocation. The small sample size limits statistical power and generalizability. However, tSCS showed consistently moderate effect sizes across all clinical assessments, indicating meaningful improvements in motor function, muscle strength, and functional independence despite the small sample size. These findings suggest clinically relevant gains even when between-group differences were not statistically significant. The relatively short intervention period may have been insufficient to achieve maximal functional improvements. Future studies with larger cohorts and extended follow-up periods are needed to better evaluate the long-term effects of tSCS in individuals with SCI. Additionally, the heterogeneity of the SCI population and the inclusion of active rehabilitation in the control group may have further reduced the ability to detect significant between-group differences.

In conclusion, repeated multisegmental tSCS appears to enhance muscle strength, functional independence, and gait function measured by the TUG in individuals with incomplete SCI, although its effects on global motor scores and basic gait measures are comparable to those of conventional rehabilitation. These results support the use of tSCS as a promising adjunct to conventional rehabilitation, particularly for targeting functional mobility and independence, with no significant adverse events reported.

## Figures and Tables

**Table 1:** Demographic data and stimulation parameters of the SCI individuals.

ID	Crossover	Group	Age	Sex	Etiology	AIS	NLI	Months Since SCI	Intensity (mA)		
									C5	L1	Coc1
Contr-1	-	Control	22	F	T	C	C8	6	-	-	-
Contr-2	-	Control	48	M	NT	D	C4	6	-	-	-
Contr-3	✓	Control	55	M	T	D	C3	5	-	-	-
Contr-4	-	Control	47	M	T	D	C4	10	-	-	-
Contr-5	-	Control	50	M	T	D	Th1	6	-	-	-
Contr-6	-	Control	44	M	NT	D	C8	25	-	-	-
Contr-7	-	Control	34	M	T	D	C5	6	-	-	-
Contr-8	✓	Control	50	M	T	D	C5	3	-	-	-
Contr-9	✓	Control	55	M	T	D	C5	4	-	-	-
Contr-10	✓	Control	62	M	T	D	C5	4	-	-	-
Mean			46.7	1F/9M	8T/2NT	1C/9D		7.5			
SD			11.4					6.4			
tSCS-1	-	tSCS	32	M	T	D	C5	4	25	44	47
tSCS-2	-	tSCS	27	M	T	D	C8	6	23	55	56
tSCS-3	✓	tSCS	55	M	T	D	C3	6	52	59	51
tSCS-4	-	tSCS	61	F	T	D	C5	72	42	48	59
tSCS-5	-	tSCS	42	M	T	D	C5	5	49	48	50
tSCS-6	-	tSCS	26	M	T	D	C6	4	38	47	54
tSCS-7	-	tSCS	26	M	T	D	C5	7	25	44	47
tSCS-8	✓	tSCS	54	M	T	D	C5	4	51	50	50
tSCS-9	✓	tSCS	55	M	T	D	C5	5	50	58	62
tSCS-10	-	tSCS	21	F	T	D	C4	4	48	66	66
tSCS-11	✓	tSCS	62	M	T	D	C5	6	50	58	62
tSCS-12	-	tSCS	41	F	T	C	C6	11	48	66	66
Mean			41.8	3F/9M	12T	1C/11D		11.2	41.8	53.6	55.8
SD			15.1					19.3	11.2	7.9	7.0
*p.* *			0.41 *	0.57 **	0.10 **	0.43 **	0.50 **	0.54 *			

M: male; F: female; T: traumatic; NT: non-traumatic; AIS: ASIA (American Spinal Injury Association) Impairment Scale; NLI: neurological level of injury; Th: thoracic; C: cervical; L: lumbar; Coc1: coccyx; ✓: Participant who crossed over to the tSCS group. *: *p*-value according to Student’s *t*-test; **: according to the chi-squared test.

**Table 2:** Median and IQR of TMS, LEMS, and SCIM-III assessments at baseline (pre), after the last session (post), and during follow-up (follow), for the control and tSCS groups.

Group		TMS			LEMS			SCIM-III		
		Pre	Post	Follow	Pre	Post	Follow	Pre	Post	Follow
Control	Median	82.5	85.5	87.0	43.5	45.0	45.0	78.5	80.0	80.0
	IQ 25–75%	66.8–90.0	68.5–91.5	70.8–92.3	29.0–48.0	31.0–48.3	32.0–48.3	69.3–87.3	68.8–93.3	68.8–92.5
tSCS	Median	84.5	86.5	87.0	43.5	45.0	46.0	78.5	83.5	84.5
	IQR 25–75%	76.0–91.0	80.5–92.8	81.3–93.0	38.0–47.3	42.0–47.5	42.0–47.8	73.3–86.0	78.3–90.0	78.3–96.0

TMS: Total Motor Score; LEMS: Lower Extremity Motor Score; SCIM-III: Spinal Cord Independence Measure Version III; IQR: interquartile range.

**Table 3:** Statistical analysis of all clinical assessments.

		Control Group						tSCS Group					
		Median	IQR 25–75%	Z Value	*p*-Value		Ef. Size	Median	IQR 25–75%	Z Value	*p*-Value		Ef. Size
				Fried.	Fried.	Wilcox.				Friedm.	Fried.	Wilcox.	
TMS	Pre	82.5	66.8–90.0	15.75	<0.001	-	-	84.5	76.0–91.0	12.67	0.002	-	-
	Post	85.5	68.5–91.5			0.007	0.57	86.5	80.5–92.8			0.012	0.54
	Follow	87.0	70.8–92.3			0.008	0.57	87.0	81.3–93.0			0.006	0.59
LEMS	Pre	43.5	29.0–48.0	12.78	0.002	-	-	43.5	38.0–47.3	15.79	<0.001	-	-
	Post	45.0	31.0–48.3			0.016	0.51	45.0	42.0–47.5			0.011	0.54
	Follow	45.0	32.0–48.3			0.016	0.51	46.0	42.0–47.8			0.007	0.58
SCIM-II	Pre	78.5	69.3–87.3	3.13	0.210	-	0.16	78.5	73.3–86.0	14.21	<0.001	-	-
	Post	80.0	68.8–93.3			-		83.5	78.3–90.0			0.018	0.51
	Follow	80.0	68.8–92.5			--		84.5	78.3–96.0			0.012	0.54
MVC-QM	Pre	16.4	11.0–26.1	3.94	0.149	-	0.20	15.8	12.8–24.7	19.54	<0.001	-	-
	Post	18.3	12.3–27.8			-		21.6	16.7–29.8			0.005	0.60
	Follow	17.0	12.2–27.9			-		22.7	18.0–30.0			0.005	0.60
MVC-TA	Pre	6.8	4.5–8.0	3.94	0.139	-	0.20	8.6	2.4–10.8	15.85	<0.001	-	-
	Post	7.5	5.0–10.4			-		11.0	7.5–12.9			0.004	0.62
	Follow	8.5	4.9–11.3			-		12.6	10.7–14.6			0.002	0.65
WISCI-II	Pre	14.0	10.5–15.5	2.00	0.368	-	0.11	15.5	13.3–18.8	6.00	0.050	-	-
	Post	14.0	11.0–15.5			-		16.0	14.3–20.0			0.109	0.34
	Follow	14.0	11.0–15.5			-		16.0	14.3–20.0			0.109	0.34
TUG	Pre	24.5	15.7–42.3	5.47	0.065	-	0.30	19.6	15.4–39.7	15.61	<0.001	-	-
	Post	21.3	14.1–35.6			-		17.1	13.7–22.9			0.003	0.64
	Follow	18.0	14.6–32.9			-		16.5	13.3–21.4			0.002	0.65
10MWT	Pre	18.6	13.2–39.1	7.82	0.020	-	-	15.7	11.4–20.1	15.87	<0.001	-	-
	Post	13.0	11.5–27.2			0.017	0.51	13.3	9.9–15.6			0.002	0.65
	Follow	13.8	11.6–28.1			0.011	0.54	12.3	9.1–15.6			0.004	0.61
6meterWT	Pre	13.4	10.0–26.8	12.00	0.002	-	-	11.5	7.7–13.3	14.60	<0.001	-	-
	Post	10.1	7.4–16.6			0.008	0.57	8.7	6.9–11.2			0.014	0.52
	Follow	9.1	7.7–12.1			0.012	0.54	9.0	6.3–10.2			0.002	0.65

TMS: Total Motor Score; LEMS: Lower Extremity Motor Score; SCIM-III: Spinal Cord Independence Measure Version III; WISCI: Walking Index for SCI; TUG: Timed Up and Go; 10MWT: 10-Meter Walking Test; 6meterWT: 6-Meter Walking Test; Fried: Friedman test used to assess differences across time points. When significant, post hoc comparisons were performed using the Wilcoxon signed-rank test, and *p*-values according to the Wilcoxon test were calculated for pre- vs. post-intervention and pre- vs. follow-up comparisons within each group. Effect sizes for pre vs. post and pre vs. follow-up: 0.2 = small, 0.5 = medium, and 0.8 = large.

**Table 4:** Median and IQR of MVC-QM and MVC-TA at baseline (pre), post-intervention (post), and during follow-up (follow), in the control and tSCS groups.

Group		MVC-QM			MVC-TA		
		Pre	Post	Follow	Pre	Post	Follow
Control	Median	16.4	18.3	17.0	6.8	7.5	8.5
	IQR 25–75%	11.0–26.1	12.3–27.8	12.2–27.9	4.5–8.0	5.0–10.4	4.9–11.3
tSCS	Median	15.8	21.6	22.7	8.6	11	12.6
	IQR 25–75%	12.8–24.7	16.7–29.8	18.0–30.0	2.4–10.8	7.5–12.9	10.7–14.6

MVC-QM: Maximum voluntary contraction of quadriceps muscle; MVC-TA: Maximum voluntary contraction of tibialis anterior muscle.

**Table 5:** Median and IQR of the WISCI-II, TUG, 10MWT, and 6meterWT at baseline (pre), post-intervention (post), and during follow-up (follow), in the control and tSCS groups.

Group		WISCI-II			TUG			10MWT			6meterWT		
		Pre	Post	Follow	Pre	Post	Follow	Pre	Post	Follow	Pre	Post	Follow
Control	Median	14.0	14.0	14.0	24.5	21.3	18.0	18.6	13.0	13.8	13.4	10.1	9.1
	IQR 25–75%	10.5–15.5	11.0–15.5	11.0–15.5	15.7–42.3	14.1–35.6	14.6–32.9	13.2–39.1	11.5–27.2	11.6–28.1	10.0–26.8	7.4–16.6	7.7–12.1
tSCS	Median	15.5	16.0	16.0	19.6	17.1	16.5	15.7	13.3	12.3	11.5	8.7	9.0
	IQR 25–75%	13.3–18.8	14.3–20.0	14.3–20.0	15.4–39.7	13.7–22.9	13.3–21.4	11.4–20.1	9.9–15.6	9.1–15.6	7.7–13.3	6.9–11.2	6.3–10.2

WISCI-II: Walking Index for SCI-II; TUG: Timed Up and Go; 10MWT: 10-Meter Walking Test; 6meterWT: 6-Meter Walking Test; IQR: interquartile range.

**Table 6:** Score changes in the control and tSCS groups at post-intervention and follow-up.

	Score Changes											
	Δ Post-Pre						Δ Follow-Pre					
	Contr.	SD	tSCS	SD	Z	*p*	Contr.	SD	tSCS	SD	Z	*p*
TMS	2.20	1.87	2.08	2.31	−0.235	0.814	3.40	3.24	2.92	2.75	−0.200	0.842
LEMS	1.30	1.25	1.92	1.88	−0.578	0.563	1.50	1.78	2.42	2.31	−1.088	0.277
SCIM-III	1.70	3.50	3.92	4.64	−1.214	0.225	1.50	3.27	5.75	7.07	−1.871	0.061
WISCI	0.33	1.00	0.92	2.11	−0.778	0.437	0.33	1.00	0.92	2.11	−0.778	0.437
TUG	−2.73	4.02	−5.22	6.39	−1.173	0.241	−4.03	6.32	−5.95	6.10	−0.782	0.434
10MWT	−4.72	5.26	−3.16	3.14	−0.107	0.915	−5.32	5.55	−4.08	4.55	−0.284	0.776
6meterWT	−5.27	4.78	−2.85	3.93	−1.387	0.166	−4.05	3.56	−3.39	4.12	−0.772	0.440
MVC-QM	1.45	4.99	4.15	4.90	−1.191	0.234	1.08	3.92	5.14	4.30	−2.046	0.041 *
MVC-TA	1.33	2.23	3.19	2.70	−1.880	0.060	1.73	2.07	5.12	3.55	−2.276	0.023 *

Contr.: control group; tSCS: tSCS group; SD: standard deviation; *p*-values according to Mann–Whitney U test: * *p* < 0.05.

## Data Availability

All of the data and materials can be found at Institut Guttmann. All of the data are expressed in the tables and figures. The datasets supporting the conclusions of this article are available in the Figshare repository under DOI https://doi.org/10.6084/m9.figshare.27118134.v1. This study was registered at ClinicalTrials.gov (Identifier: NCT07289191).

## Supplementary Materials

The following supporting information can be downloaded at https://www.mdpi.com/article/10.3390/biomedicines14061214/s1: Table S1: TMS, LEMS and SCIM-III assessments at baseline (pre), after the last session (post) and during follow-up (follow) for each individual in the control and tSCS groups; Table S2: MVC-QM and MVC-TA at baseline (pre), post intervention (post) and during follow-up (follow) for each individual in the control and tSCS groups; Table S3: Gait assessment using WISCI-II, TUG, 10MWT, 6meterWT at baseline (pre), post intervention (post) and during follow-up (follow) for each individual in the control and tSCS groups.

## Abbreviations

The following abbreviations are used in this manuscript (in order of appearance):

tSCS	Transcutaneous Spinal Cord Stimulation
SCI	Spinal Cord Injury
AIS	American Spinal Injury Association Impairment Scale
TMS	Total Motor Score
LEMS	Lower Extremity Motor Score
WISCI-II	Walking Index for Spinal Cord Injury—II
10MWT	10-Meter Walking Test
6meterWT	6-Meter Walking Test
TUG	Timed Up and Go
MVC	Maximal Voluntary Contraction
QM	Quadriceps
TA	Tibialis Anterior
SCIM-III	Spinal Cord Independence Measure—III
SCS	Spinal Cord Stimulation
T11	11th Thoracic Vertebra
Cocc1	1st Coccygeal Vertebra
MAS	Modified Ashworth Scale
ASIA	American Spinal Injury Association
MVC-QM	Maximal Voluntary Contraction of the Quadriceps
MVC-TA	Maximal Voluntary Contraction of the Tibialis Anterior
EMG	Electromyography
IQR	Interquartile Range
NLI	Neurological Level of Injury
C	Cervical
L	Lumbar
Cocc	Coccyx
F	Female
M	Male
T	Traumatic
NT	Non-Traumatic
Th	Thoracic

## References

[1] Safdarian M., Trinka E., Rahimi-Movaghar V., Thomschewski A., Aali A., Abady G.G., Abate S.M., Abd-Allah F., Abedi A., Adane D.E. (2023). Global, Regional, and National Burden of Spinal Cord Injury, 1990–2019: A Systematic Analysis for the Global Burden of Disease Study 2019. Lancet Neurol..

[2] Crispo J.A.G., Kuramoto L.K., Cragg J.J. (2023). Global Burden of Spinal Cord Injury: Future Directions. Lancet Neurol..

[3] Ditunno P.L., Patrick M., Stineman M., Ditunno J.F. (2008). Who Wants to Walk? Preferences for Recovery after SCI: A Longitudinal and Cross-Sectional Study. Spinal Cord.

[4] Fouad K., Pearson K. (2004). Restoring Walking after Spinal Cord Injury. Prog. Neurobiol..

[5] Awai L., Curt A. (2015). Comprehensive Assessment of Walking Function after Human Spinal Cord Injury. Prog. Brain Res..

[6] Hofstoetter U.S., Krenn M., Danner S.M., Hofer C., Kern H., McKay W.B., Mayr W., Minassian K. (2015). Augmentation of Voluntary Locomotor Activity by Transcutaneous Spinal Cord Stimulation in Motor-Incomplete Spinal Cord-Injured Individuals. Artif. Organs.

[7] Estes S., Zarkou A., Hope J.M., Suri C., Field-Fote E.C. (2021). Combined Transcutaneous Spinal Stimulation and Locomotor Training to Improve Walking Function and Reduce Spasticity in Subacute Spinal Cord Injury: A Randomized Study of Clinical Feasibility and Efficacy. J. Clin. Med..

[8] Hofstoetter U.S., Freundl B., Lackner P., Binder H. (2021). Transcutaneous Spinal Cord Stimulation Enhances Walking Performance and Reduces Spasticity in Individuals with Multiple Sclerosis. Brain Sci..

[9] Kumru H., Ros-Alsina A., García Alén L., Vidal J., Gerasimenko Y., Hernandez A., Wrigth M. (2024). Improvement in Motor and Walking Capacity during Multisegmental Transcutaneous Spinal Stimulation in Individuals with Incomplete Spinal Cord Injury. Int. J. Mol. Sci..

[10] Kumru H., García-Alén L., Ros-Alsina A., Albu S., Valles M., Vidal J. (2023). Transcutaneous Spinal Cord Stimulation Improves Respiratory Muscle Strength and Function in Subjects with Cervical Spinal Cord Injury: Original Research. Biomedicines.

[11] Gerasimenko Y., Gorodnichev R., Puhov A., Moshonkina T., Savochin A., Selionov V., Roy R.R., Lu D.C., Edgerton V.R. (2015). Initiation and Modulation of Locomotor Circuitry Output with Multisite Transcutaneous Electrical Stimulation of the Spinal Cord in Noninjured Humans. J. Neurophysiol..

[12] Gerasimenko Y.P., Lu D.C., Modaber M., Zdunowski S., Gad P., Sayenko D.G., Morikawa E., Haakana P., Ferguson A.R., Roy R.R. (2015). Noninvasive Reactivation of Motor Descending Control after Paralysis. J. Neurotrauma.

[13] Minassian K., Hofstoetter U.S., Danner S.M., Mayr W., Bruce J.A., McKay W.B., Tansey K.E. (2016). Spinal Rhythm Generation by Step-Induced Feedback and Transcutaneous Posterior Root Stimulation in Complete Spinal Cord–Injured Individuals. Neurorehabil. Neural Repair.

[14] Murray L.M., Knikou M. (2019). Transspinal Stimulation Increases Motoneuron Output of Multiple Segments in Human Spinal Cord Injury. PLoS ONE.

[15] Gad P., Gerasimenko Y., Zdunowski S., Turner A., Sayenko D., Lu D.C., Edgerton V.R. (2017). Weight Bearing Over-Ground Stepping in an Exoskeleton with Non-Invasive Spinal Cord Neuromodulation after Motor Complete Paraplegia. Front. Neurosci..

[16] García-Alén L., Kumru H., Castillo-Escario Y., Benito-Penalva J., Medina-Casanovas J., Gerasimenko Y.P., Edgerton V.R., García-Alías G., Vidal J. (2023). Transcutaneous Cervical Spinal Cord Stimulation Combined with Robotic Exoskeleton Rehabilitation for the Upper Limbs in Subjects with Cervical SCI: Clinical Trial. Biomedicines.

[17] Inanici F., Samejima S., Gad P., Edgerton V.R., Hofstetter C.P., Moritz C.T. (2018). Transcutaneous Electrical Spinal Stimulation Promotes Long-Term Recovery of Upper Extremity Function in Chronic Tetraplegia. IEEE Trans. Neural Syst. Rehabil. Eng..

[18] McPherson J.G., Miller R.R., Perlmutter S.I. (2015). Targeted, Activity-Dependent Spinal Stimulation Produces Long-Lasting Motor Recovery in Chronic Cervical Spinal Cord Injury. Proc. Natl. Acad. Sci. USA.

[19] Sayenko D.G., Rath M., Ferguson A.R., Burdick J.W., Havton L.A., Edgerton V.R., Gerasimenko Y.P. (2019). Self-Assisted Standing Enabled by Non-Invasive Spinal Stimulation after Spinal Cord Injury. J. Neurotrauma.

[20] Rath M., Vette A.H., Ramasubramaniam S., Li K., Burdick J., Edgerton V.R., Gerasimenko Y.P., Sayenko D.G. (2018). Trunk Stability Enabled by Noninvasive Spinal Electrical Stimulation after Spinal Cord Injury. J. Neurotrauma.

[21] Kumru H., Flores Á., Rodríguez-Cañón M., Edgerton V.R., García L., Benito-Penalva J., Navarro X., Gerasimenko Y., García-Alías G., Vidal J. (2021). Cervical Electrical Neuromodulation Effectively Enhances Hand Motor Output in Healthy Subjects by Engaging a Use-Dependent Intervention. J. Clin. Med..

[22] Kumru H., Rodríguez-Cañón M., Edgerton V.R., García L., Flores Á., Soriano I., Opisso E., Gerasimenko Y., Navarro X., García-Alías G. (2021). Transcutaneous Electrical Neuromodulation of the Cervical Spinal Cord Depends Both on the Stimulation Intensity and the Degree of Voluntary Activity for Training. A Pilot Study. J. Clin. Med..

[23] Comino-Suárez N., Moreno J.C., Megía-García Á., del-Ama A.J., Serrano-Muñoz D., Avendaño-Coy J., Gil-Agudo Á., Alcobendas-Maestro M., López-López E., Gómez-Soriano J. (2025). Transcutaneous Spinal Cord Stimulation Combined with Robotic-Assisted Body Weight-Supported Treadmill Training Enhances Motor Score and Gait Recovery in Incomplete Spinal Cord Injury: A Double-Blind Randomized Controlled Clinical Trial. J. Neuroeng. Rehabil..

[24] Hofstoetter U.S., Hofer C., Kern H., Danner S.M., Mayr W., Dimitrijevic M.R., Minassian K. (2013). Effects of Transcutaneous Spinal Cord Stimulation on Voluntary Locomotor Activity in an Incomplete Spinal Cord Injured Individual. Biomed. Eng. Biomed. Tech..

[25] McHugh L.V., Miller A.A., Leech K.A., Salorio C., Martin R.H. (2020). Feasibility and Utility of Transcutaneous Spinal Cord Stimulation Combined with Walking-Based Therapy for People with Motor Incomplete Spinal Cord Injury. Spinal Cord Ser. Cases.

[26] Moritz C., Field-Fote E.C., Tefertiller C., van Nes I., Trumbower R., Kalsi-Ryan S., Purcell M., Janssen T.W.J., Krassioukov A., Morse L.R. (2024). Non-Invasive Spinal Cord Electrical Stimulation for Arm and Hand Function in Chronic Tetraplegia: A Safety and Efficacy Trial. Nat. Med..

[27] Angeli C.A., Gerasimenko Y. (2023). Combined Cervical Transcutaneous with Lumbosacral Epidural Stimulation Improves Voluntary Control of Stepping Movements in Spinal Cord Injured Individuals. Front. Bioeng. Biotechnol..

[28] Benavides F.D., Jo H.J., Lundell H., Edgerton V.R., Gerasimenko Y., Perez M.A. (2020). Cortical and Subcortical Effects of Transcutaneous Spinal Cord Stimulation in Humans with Tetraplegia. J. Neurosci..

[29] Barss T.S., Parhizi B., Mushahwar V.K. (2020). Transcutaneous Spinal Cord Stimulation of the Cervical Cord Modulates Lumbar Networks. J. Neurophysiol..

[30] McCrea D.A., Rybak I.A. (2008). Organization of Mammalian Locomotor Rhythm and Pattern Generation. Brain Res. Rev..

[31] Whelan P., Bonnot A., O’Donovan M.J. (2000). Properties of Rhythmic Activity Generated by the Isolated Spinal Cord of the Neonatal Mouse. J. Neurophysiol..

[32] Etlin A., Blivis D., Ben-Zwi M., Lev-Tov A. (2010). Long and Short Multifunicular Projections of Sacral Neurons Are Activated by Sensory Input to Produce Locomotor Activity in the Absence of Supraspinal Control. J. Neurosci..

[33] Kirshblum S.C., Burns S.P., Biering-Sorensen F., Donovan W., Graves D.E., Jha A., Johansen M., Jones L., Krassioukov A., Mulcahey M.J. (2011). International Standards for Neurological Classification of Spinal Cord Injury (Revised 2011). J. Spinal Cord Med..

[34] van Hedel H.J., Wirz M., Dietz V. (2005). Assessing Walking Ability in Subjects with Spinal Cord Injury: Validity and Reliability of 3 Walking Tests. Arch. Phys. Med. Rehabil..

[35] Shapkova E.Y., Pismennaya E.V., Emelyannikov D.V., Ivanenko Y. (2020). Exoskeleton Walk Training in Paralyzed Individuals Benefits From Transcutaneous Lumbar Cord Tonic Electrical Stimulation. Front. Neurosci..

