# Supplementary material for: Changes in Motor, Functional Independence, and Gait Recovery After Incomplete Spinal Cord Injury with Transcutaneous Spinal Cord Stimulation: A Randomized Controlled Trial with a Partial Crossover Design
Source: Biomedicines. 2026 May 27;14(6):1214. doi: 10.3390/biomedicines14061214 (PMC13296979; doi:10.3390/biomedicines14061214)
Supplement: Supplementary file 1 [file biomedicines-14-01214-s001.zip › biomedicines-4301501-supplementary.pdf]

biomedicines

MDPI

Supplementary Materials

Changes in Motor, Functional Independence, and Gait Recovery After Incomplete Spinal Cord Injury with Transcutaneous Spinal Cord Stimulation: A Randomized Controlled Trial with a Partial Crossover Design

Hatice Kumru, Aina Ros-Alsina, Agustín Hernandez-Navarro, Eloy Opisso, Margarita Valles, Jesus Benito-Penalva, Joan Vidal, Miquel Sarrio and Loreto García Alén

Table S1. TMS, LEMS and SCIM-III assessments at baseline (pre), after the last session (post) and during follow-up (follow) for each individual in the control and tSCS groups.

Subject	TMS			LEMS			SCIM-III		
	Pre	Post	Follow	Pre	Post	Follow	Pre	Post	Follow
Contr-1	57	57	57	12	12	12	71	70	70
Contr-2	94	95	95	47	48	48	88	94	94
Contr-3	90	91	91	50	50	50	100	100	100
Contr-4	56	58	67	26	28	32	48	49	48
Contr-5	70	72	72	30	32	32	74	74	74
Contr-6	88	89	91	44	45	45	83	93	92
Contr-7	90	91	92	48	49	49	87	87	87
Contr-8	78	82	83	41	45	43	73	73	73
Contr-9	72	76	75	43	45	45	64	65	65
Contr-10	87	93	93	48	48	48	86	86	86
Median	82.5	85.5	87.0	43.5	45.0	45.0	78.5	80.0	80.0
IQ 25-75%	66.8-90.0	68.5-91.5	70.8-92.3	29.0-48.0	31.0-48.3	32.0-48.3	69.3-87.3	68.8-93.3	68.8-92.5
tSCS-1	92	93	93	48	48	48	86	86	86
tSCS-2	88	92	92	38	42	42	79	79	79
tSCS-3	91	91	93	50	50	50	100	100	100
tSCS-4	81	85	85	38	42	42	82	82	84
tSCS-5	76	83	85	38	43	46	69	80	81
tSCS-6	91	93	93	45	46	46	92	93	93
tSCS-7	86	88	89	44	46	46	77	85	85
tSCS-8	83	82	82	43	45	45	73	78	78

tSCS-9	75	77	81	45	45	47	65	69	71
tSCS-10	73	73	74	39	40	41	74	87	97
tSCS-11	93	93	93	48	48	48	86	91	98
tSCS-12	76	80	80	28	32	32	78	78	78
Median	84.5	86.5	87.0	43.5	45.0	46.0	78.5	83.5	84.5
IQR 25-75%	76.0-91.0	80.5-92.8	81.3-93.0	38.0-47.3	42.0-47.5	42.0-47.8	73.3-86.0	78.3-90.0	78.3-96.0

TMS: Total motor score; LEMS: Lower extremity motor score; SCIM-III: Spinal Cord Independence Measure Version III; IQR: interquartile range.

Table S2. MVC-QM and MVC-TA at baseline (pre), post intervention (post) and during follow-up (follow) for each individual in the control and tSCS groups.

Subject	MVC-QM			MVC-TA		
	Pre	Post	Follow	Pre	Post	Follow
Contr-01	0	0	1	7.8	6.5	7.3
Contr-02	11.8	25	20.5	3.1	5	4.9
Contr-03	26.5	30	30	13	12.9	11.2
Contr-04	18.4	12.7	12.5	2.8	4.2	4.9
Contr-05	14.3	11.2	11.2	4.9	4.9	4.9
Contr-06	25.9	27	27.2	7.3	13.8	11.5
Contr-07	30	30	30	7.9	8.4	8.6
Contr-08	9.8	13.4	13.4	6.2	9.6	9.6
Contr-09	11.4	12.8	12.6	5	6	8.4
Contr-10	22.7	23.2	23.2	8.4	8.4	12.4
Median	16.4	18.3	17.0	6.8	7.5	8.5
IQR 25-75%	11.0-26.1	12.3-27.8	12.2-27.9	4.5-8.0	5.0-10.4	4.9-11.3
tSCS-1	15.6	23.5	22.4	7.6	9.7	14.9
tSCS-2	7.3	7.5	9.4	0.6	2.6	2.8
tSCS-3	30	30	30	11.2	13	17.1
tSCS-4	6.8	8.7	12.4	0.3	5.6	6.1
tSCS-5	17.1	18.1	22.9	8.4	10.3	10.3
tSCS-6	27.2	29.1	29.9	11.5	11.8	12

tSCS-7	30	30	30	9.5	12.7	13.5
tSCS-8	13.4	26	25.5	9.6	12.9	13.3
tSCS-9	12.6	19.6	19.3	8.4	8	12.2
tSCS-10	14.2	17.4	18.7	0.6	7.3	13
tSCS-11	16.5	30	30	12.4	21.6	23.1
tSCS-12	15.9	16.5	17.8	8.8	11.7	12
Median	15.8	21.6	22.7	8.6	11	12.6
IQR 25-75%	12.8-24.7	16.7-29.8	18.0-30.0	2.4-10.8	7.5-12.9	10.7-14.6

MVC-QM: Maximum voluntary contraction of quadriceps muscle; MVC-TA: Maximum voluntary contraction of tibialis anterior muscle

Table S3. Gait assessment using WISCI-II, TUG, 10MWT, 6meterWT at baseline (pre), post intervention (post) and during follow-up (follow) for each individual in the control and tSCS groups.

Subject	WISCI-II			TUG			10MWT			6meterWT		
	Pre	Post	Follow	Pre	Post	Follow	Pre	Post	Follow	Pre	Post	Follow
Contr-1	-	-	-	-	-	-	-	-	-	-	-	-
Contr-2	15	15	15	21.5	20.2	18.0	14.1	12.2	12.1	13.4	7.7	8.2
Contr-3	20	20	20	10.2	9.7	10.8	8.3	8.6	8.5	7.1	5.5	5.1
Contr-4	9	9	9	65.0	68.0	70.0	55	55	48.2	44	32.3	33.1
Contr-5	8	8	8	49.5	44.8	44.8	44.8	35.5	35.5	36	21.8	NA
Contr-06	12	15	15	14.8	12.5	13.8	12.3	11.4	11	9.4	7	7.6
Contr-07	16	16	16	24.5	24.5	19.6	18.6	11.5	13.8	10.5	9.7	8.1
Contr-08	14	14	14	16.6	15.6	15.3	25.3	18.7	20.7	13.6	10.1	12.5
Contr-09	14	14	14	35.0	26.3	17.9	33.3	18	15.5	17.5	11.3	9.9
Contr-10	13	13	13	30.3	21.3	21.0	14.7	13	13.2	12.1	10.8	10.7
Median	14.0	14.0	14.0	24.5	21.3	18.0	18.6	13.0	13.8	13.4	10.1	9.1
IQR 25-75%	10.5-15.5	11.0-15.5	11.0-15.5	15.7-42.3	14.1-35.6	14.6-32.9	13.2-39.1	11.5-27.2	11.6-28.1	10.0-26.8	7.4-16.6	7.7-12.1
tSCS-1	20	20	20	17.1	14.4	13.0	10.5	8.4	8.2	6.1	6.6	6.0
tSCS-2	18	18	18	15.7	13.6	14.0	12.4	11.3	9.0	7.8	7.8	7.1
tSCS-3	20	20	20	10.8	7.9	8.1	8.5	7.3	6.6	5.1	4.6	4.4
tSCS-4	16	16	16	41.7	38.5	38.3	32.0	31.1	32.1	23.8	18.5	18.3
tSCS-5	16	16	16	42.8	19.9	21.8	15.8	13.5	15.8	13.5	8.4	9.0

tSCS-6	15	15	15	13.8	11.1	11.1	11.0	9.4	9.3	7.6	5.7	5.2
tSCS-7	13	16	16	33.7	23.6	20.3	18.4	13.0	12.5	12.4	8.9	7.2
tSCS-8	14	14	14	15.3	14.0	14.8	20.7	15.6	14.8	12.5	10.0	10.3
tSCS-9	14	14	14	17.9	18.6	16.8	15.5	13.9	12.9	9.9	11.0	9.5
tSCS-10	13	20	20	29.5	20.7	18.7	17.6	15.4	12.1	12.2	11.2	9.0
tSCS-11	19	20	20	21.2	15.6	16.2	15.0	12.6	12.1	10.7	8.2	9.9
tSCS-12	8	8	8	93.0	92.0	88.0	79.0	67.0	62.0	66.0	52.5	51.0
Median	15.5	16.0	16.0	19.6	17.1	16.5	15.7	13.3	12.3	11.5	8.7	9.0
IQR 25-75%	13.3-18.8	14.3-20.0	14.3-20.0	15.4-39.7	13.7-22.9	13.3-21.4	11.4-20.1	9.9-15.6	9.1-15.6	7.7-13.3	6.9-11.2	6.3-10.2

WISCI-II: Walking index for SCI-II; TUG: Time Up and Go; 10MWT: 10-Meter Walking Test; 6meterWT: 6-Meter Walking Test; NA: Not available.; IQR: interquartile range.
